# Bleeding and first-year mortality following hip fracture surgery and preoperative use of low-dose acetylsalicylic acid: an observational cohort study

**DOI:** 10.1186/1471-2474-12-254

**Published:** 2011-11-07

**Authors:** Annika M Kragh, Markus Waldén, Anna Apelqvist, Philippe Wagner, Isam Atroshi

**Affiliations:** 1 Department of Orthopedics, Hässleholm Hospital, SE-281 25 Hässleholm, Sweden; 2 Institute of Health Sciences, Department of Geriatrics, Lund University, Lund, Sweden; 3 Department of Medical and Health Sciences, Linköping University, Linköping, Sweden; 4 National Competence Center for Musculoskeletal Disorders, Department of Orthopedics, Lund University, Lund, Sweden; 5 Department of Clinical Sciences Lund, Lund University, Lund, Sweden

## Abstract

**Background:**

Hip fracture is associated with high mortality. Cardiovascular disease and other comorbidities requiring long-term anticoagulant medication are common in these mostly elderly patients. The objective of our observational cohort study of patients undergoing surgery for hip fracture was to study the association between preoperative use of low-dose acetylsalicylic acid (LdAA) and intraoperative blood loss, blood transfusion and first-year all-cause mortality.

**Methods:**

An observational cohort study was conducted on patients with hip fracture (cervical requiring hemiarthroplasty or pertrochanteric or subtrochanteric requiring internal fixation) participating in a randomized trial that found lack of efficacy of a compression bandage in reducing postoperative bleeding. The participants were 255 patients (≥50 years) of whom 118 (46%) were using LdAA (defined as ≤320 mg daily) preoperatively. Bleeding variables in patients with and without LdAA treatment at time of fracture were measured and blood transfusions given were compared using logistic regression. The association between first-year mortality and preoperative use of LdAA was analyzed with Cox regression adjusting for age, sex, type of fracture, baseline renal dysfunction and baseline cardiovascular and/or cerebrovascular disease.

**Results:**

Blood transfusions were given postoperatively to 74 (62.7%) LdAA-treated and 76 (54%) non-treated patients; the adjusted odds ratio was 1.8 (95% CI 1.04 to 3.3). First-year mortality was significantly higher in LdAA-treated patients; the adjusted hazard ratio (HR) was 2.35 (95% CI 1.23 to 4.49). The mortality was also higher with baseline cardiovascular and/or cerebrovascular disease, adjusted HR 2.78 (95% CI 1.31 to 5.88). Patients treated with LdAA preoperatively were significantly more likely to suffer thromboembolic events (5.7% vs. 0.7%, P = 0.03).

**Conclusions:**

In patients with hip fracture (cervical treated with hemiarthroplasty or pertrochanteric or subtrochanteric treated with internal fixation) preoperative use of low-dose acetylsalicylic acid was associated with significantly increased need for postoperative blood transfusions and significantly higher all-cause mortality during one year after surgery.

## Background

Hip fracture is common in elderly people and these patients have a more than doubled mortality risk compared to that of an age-matched non-fracture population [1]. Large studies have reported 30-day mortality of 6% to 11% and a 90-day mortality of up to 20% [2,3]. The causes of the high mortality are not fully known and although comorbidities, including cardiovascular disease, have been suggested to at least partly explain the increased mortality, other factors may be involved [4,5]. Since antiplatelet drugs and anticoagulants are increasingly used for primary and secondary prevention in cardiovascular disease, a large proportion of patients admitted for hip fracture are on such treatment, mainly low-dose acetylsalicylic acid (LdAA). Recent studies have recommended the continuation over surgery for many of these agents with the exception of warfarin and other vitamin K antagonists [6-8]. The rationale for not discontinuing LdAA prior to emergency fracture surgery is its irreversible inhibition of platelet function for the platelets' entire life span (8 to 10 days). However, possible association between preoperative anti-platelet therapy, specifically LdAA, and mortality following hip fracture has not previously been ascertained. We performed a randomized controlled trial to assess the efficacy of a pneumatic compression bandage, applied to the hip immediately after hip fracture surgery, in reducing the need for blood transfusion and found that the bandage did not reduce the proportion of transfused patients or the amount of transfusion [9]. In this observational study of the trial participants we analyzed intraoperative blood loss, transfusions, postoperative complications and first-year all-cause mortality after surgery. Our hypothesis was that patients using LdAA before the hip fracture had higher need for blood transfusions and higher first-year mortality than those not using LdAA at the time of hip fracture.

## Methods

The original randomized trial has been described in detail elsewhere [9]. The aim of the trial was to evaluate the efficacy if a pneumatic compression bandage applied over the hip after surgery for a hip fracture. Briefly, patients with a proximal femoral fracture presenting at the Emergency Department of Kristianstad Hospital, Sweden, January 2005 through December 2006, were screened by an orthopedic surgeon for enrollment in the trial. The inclusion criteria were patients 50 years or older with cervical fractures planned for hemiarthroplasty or pertrochanteric or subtrochanteric fractures planned for internal fixation with plate and gliding screw or twin hook or with proximal intramedullary nail. The exclusion criteria were non-displaced subcapital (intracapsular) cervical fracture planned for internal fixation, pathologic fracture due to malignancy, concomitant fractures or injuries that might require blood transfusion, and patients refusing blood transfusion. Patients were randomly assigned to the compression group (n = 136) or non-compression group (n = 152) immediately after surgery. The results of the trial showed that the compression bandage did not have any effect on bleeding or blood transfusion requirements. For the present report focusing on preoperative use of LdAA, patients from both arms of the original trial were combined as we did not anticipate that the compression bandage had any bearing on the results.

### Treatment

According to clinical practice prophylaxis against perioperative bleeding was given to all patients with an intravenous injection of tranexamic acid (100 mg/kg body weight) 20 minutes preoperatively and a second injection after 4 hours. Prophylactic treatment against thromboembolic events was given to all patients with a subcutaneous injection of 40 mg enoxaparin daily for at least 10 days after surgery. The transfusion threshold used was blood hemoglobin (Hb) below 100 g/L.

Patients on LdAA (defined as 320 mg daily or lower), dipyridamol or clopidogrel continued with the medication, but patients on warfarin had their medication withdrawn on admission and were managed according to vitamin K antagonist reversing strategy.

### Assessments

At baseline, preoperative use of medication with antithrombotic effect, including warfarin, acetylsalicylic acid or other anti-platelet drugs, and non-steroidal anti-inflammatory drugs (NSAIDs), were recorded. At admission, Hb, platelet count, international normalized ratio (INR), activated partial thromboplastin time (APTT), and serum creatinine, were measured. The examining physician obtained medical history and recorded the presence or absence of specific conditions on a preoperative standardized protocol; heart disease (without further specification), hypertension, diabetes, lung disease (specified as asthma, chronic bronchitis or other pulmonary disease), rheumatoid arthritis, and other serious disease. These diagnoses were then verified through review of patients' records by one investigator (AK) who also documented the presence of cardiovascular disease, cerebrovascular disease or both. Before surgery, the anesthesiologist classified the patients according to the American Society of Anesthesiologists (ASA) score.

Intraoperative blood loss was estimated by the nurse anesthetist according to standard procedures. The number of red cell units transfused before, during and after surgery (up to discharge from hospital or death) was recorded. Complications up to 3 months after surgery were recorded. First-year deaths from any cause and the date of death were retrieved from the hospital administrative database linked to the National Board of Health and Welfare's Cause of Death Register.

### Sample size

The pretrial sample size estimation was based on the original trial's two transfusion-related primary outcome measures [9]. As a measure of precision in this analysis we present 95% confidence intervals for the differences in bleeding and transfusion-related variables and mortality.

### Ethics

The study was approved by the Medical Research Ethics Committee of Lund University (704/2004-11-30). Informed consent was obtained from all participating patients or, for patients who could not provide consent themselves due to cognitive impairment, from a family member.

### Statistical analysis

Baseline characteristics of the patients using and those not using LdAA preoperatively were compared with the t-test for continuous variables and Fisher's exact test for proportions. Bleeding and transfusion-related variables were compared between the two groups using logistic regression or analysis of covariance (ANCOVA) adjusting for age (as continuous variable), sex, baseline Hb and type of surgery and odds ratios and 95% confidence intervals (CI) were calculated. A Kaplan-Meier survival curve was constructed to compare first-year mortality among patients according to preoperative use of LdAA. The Fisher exact test was used to compare mortality according to patient sex and dose of preoperative LdAA. The Fisher exact test was also used to compare postoperative complications in LdAA users and non-users. A Cox regression analysis was performed with first-year mortality as the dependent variable. The independent variables were age, sex, LdAA at the time of fracture, type of fracture, baseline cardiovascular and/or cerebrovascular disease and renal dysfunction. The hazard ratio (HR) with 95% CI was calculated. Because patients with hypertension were classified as having a cardiovascular disease and the ASA score (I, II or III/IV) was essentially based on cardiovascular disease we did not include these two variables in the model. To investigate whether a relationship between preoperative use of LdAA and mortality may differ between women and men we repeated the Cox regression analysis adding a term for the interaction between sex and preoperative LdAA use to the model. Adding compression use as a covariate in the models did not change the results. All analyses were two-sided and a P value < 0.05 was considered to indicate statistical significance.

## Results

### Patients

During the study period 555 consecutive patients with potentially eligible hip fracture were registered at the emergency department. Of 333 patients assessed for eligibility by the orthopedic surgeon on duty at the Emergency Department, 288 patients were included in the original randomized trial (14 did not meet the inclusion criteria, 9 refused to participate, 4 had a refracture and 18 were excluded for other reasons). Of the 222 patients not assessed for eligibility (mean age 83 years), 70% were women, and 51% had a cervical and 49% a pertrochanteric or a subtrochanteric fracture.

The number of patients excluded from our present analysis was 33; patients receiving warfarin (n = 24) and patients receiving high-dose acetylsalicylic acid, dipyridamol or clopidogrel (n = 9). Of the 255 trial participants eligible for this analysis, 118 (46.3%) used LdAA (50 mg in 1 patient, 75 mg in 68 patients, 150 mg in 2 patients, 160 mg in 40 patients, 250 mg in 2 patients and 320 mg in 5 patients). Patients using LdAA preoperatively were older and more likely to have hypertension, cardiovascular and/or cerebrovascular disease, renal dysfunction and ASA score of III or IV than patients not using LdAA (Table 1).

**Table 1:** Characteristics of the patients according to preoperative use of low-dose acetylsalicylic acid (LdAA)

	LdAA	No LdAA	P value
	(n = 118)	(n = 137)	
Age, mean (SD)	84.0 (7.6)	80.8 (9.5)	< 0.01
Women, n (%)	82 (69.5)	108 (78.8)	0.11
Institutional residence, n (%)	29 (24.6)	31 (22.6)	0.77
Type of surgery (fracture), n (%)			0.07
Hemiarthroplasty (cervical)	51 (43.2)	43 (31.4)*	
Fixation (subtrochanteric or pertrochanteric)	67 (56.8)	94 (68.6)	
Time from admission to surgery (hrs)^†^, mean (SD)	20.1 (10.7)	19.2 (9.3)	0.51
Type of anesthesia, n (%)			
Spinal	113 (95.8)	136 (99.3)	0.1
General	5 (4.2)	1 (0.7)	0.1
NSAID medication at fracture, n (%)	6 (5.1)	8 (5.8)	1.00
Medical history, n (%)			
Cardiovascular disease	72 (61)	56 (41)	< 0.01
Cerebrovascular disease	16 (13.6)	3 (2.2)	< 0.01
Hypertension	46 (39)	33 (24.1)	0.01
Diabetes	17 (14.4)	12 (8.8)	0.17
Renal dysfunction (serum creatinine)‡	31 (26.3)	15 (11.0)	< 0.01
Pulmonary disease	8 (6.8)	15 (10.9)	0.28
Rheumatoid arthritis	2 (1.7)	8 (5.8)	0.11
Other disease	2 (1.7)	2 (1.5)	1.00
ASA score n (%)			0.02
I	7 (5.9)	15 (10.9)	
II	54 (45.8)	81 (59.1)	
III	52 (44.1)	38 (27.7)	
IV	5 (4.2)	3 (2.2)	
Compression bandage, n (%)	55 (47)	59 (43)	0.61

*Including 1 total arthroplasty

^† ^No data available for 15 patients receiving and 10 patients not receiving acetylsalicylic acid

‡ Defined as creatinine ≥105 μmol/L in males and ≥95 μmol/L in females

ASA: American Society of Anesthesiologists; NSAID: non-steroidal anti-inflammatory drugs.

### Bleeding and Transfusion

Patients using LdAA at the time of fracture had significantly higher APTT and INR values and were significantly more likely to receive postoperative blood transfusion (odds ratio 1.8), but did not differ significantly from patients not using LdAA regarding the proportion transfused or the amount of blood transfusions given before and during surgery (Table 2).

**Table 2:** Bleeding and transfusion-related data of the patients according to preoperative use of low-dose acetylsalicylic acid (LdAA)

	LdAA	No LdAA	Mean difference or odds ratio (95% CI) *	P value
	(n = 118)	(n = 137)		
Hemoglobin (g/L) at baseline	126 (15.8)	124 (14.2)		0.16
Platelet count (×10^9/L^)	264 (125)	263 (90)		0.97
APTT	33.1 (5.9)	31.6 (4.1)		0.02
INR	1.07 (0.12)	1.04 (0.09)		0.01
Patients transfused pre- or intraoperatively	47 (39.8)	49 (35.8)	1.5 (0.78, 2.8)	0.23
Units transfused pre- or intraoperatively	0.7 (0.9)	0.7 (1.0)	0.1 (-0.1, 0.3)	0.27
Bleeding intraoperatively (mL)	363 (235)	316 (329)	45 (-27, 117)	0.22
Patients transfused postoperatively	74 (62.7)	76 (54)	1.8 (1.04, 3.3)	0.04
Units transfused postoperatively	1.3 (1.3)	1.1 (1.2)	0.3 (-0.01, 0.62)	0.06
Hemoglobin day 1 (g/L)	109 (11.1)	108 (10.4)	0.3 (-2.4, 3.0)	0.89
Hemoglobin day 5 (g/L)^† ^	111 (10.7)	113 (9.5)	-1.9 (-4.5, 0.77)	0.11

Values are shown as mean (SD) except patients transfused shown as n (%)

*Logistic regression or ANCOVA adjusting for age, sex, baseline hemoglobin and type of surgery.

^† ^No data were available for 7 patients using and 5 patients not using acetylsalicylic acid

APTT: activated partial thromboplastin time; INR: international normalized ratio

### Mortality

**Figure 1:**
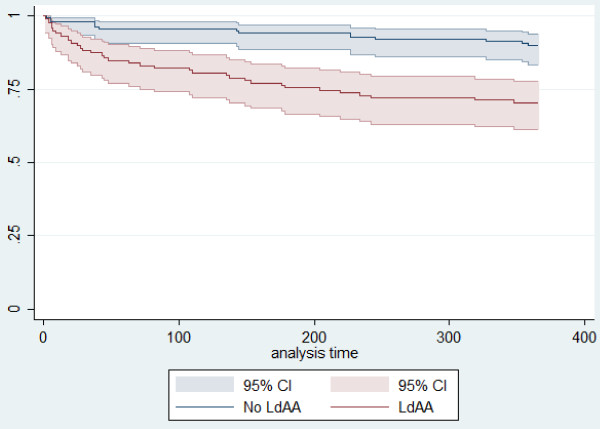
**Kaplan-Meier survival estimates during first year after surgery for hip fracture according to preoperative use of low-dose acetylsalicylic acid (LdAA), analysis time is number of days since surgery**.

At 30 days after surgery 17 patients (6.7%) had died, 14 were preoperative LdAA users and 3 were non-users (11.9% versus 2.2%). The 90-day mortality was 10.6% and first-year mortality was 19.2%, both higher among preoperative LdAA users (17.8% vs. 4.4% and 29.7% vs. 10.2% respectively) (Figure 1). Among the preoperative LdAA users the type of surgery was hemiarthroplasty in 14 of the 35 1-year non-survivors (40%) and in 37 of the 83 survivors (45%). Among the preoperative LdAA users, 1-year mortality was 31.7% (26 of 82) in women and 25% (9 of 36) in men (P = 0.52) and among non-users it was 9.3% (10 of 108) in women and 13.8% (4 of 29) in men (P = 0.50). Among the LdAA users treated with ≤75 mg, 1-year mortality was 30.4% (21 of 69 patients) and among those treated with ≥150 mg it was 28.6% (14 of 49 patients) (P = 1.0).

In the Cox regression analysis use of LdAA preoperatively was associated with significantly higher first-year mortality, with an adjusted HR of 2.35 (Table 3). In the Cox regression model that included a term for the interaction between sex and preoperative LdAA use, the sex-specific HR was estimated to be 2.88 for women and 1.25 for men. However, this difference in effect of LdAA was shown not to be statistically significant when tested using a likelihood-ratio test (P = 0.25).

**Table 3:** First-year mortality hazard ratios for baseline factors including preoperative use of low-dose acetylsalicylic acid (LdAA) in a Cox regression

Factors	Hazard ratio	95% CI	P value
Preoperative LdAA use	2.35	1.23 - 4.49	0.01
Age at fracture (per year)	1.08	1.03 - 1.13	< 0.01
Sex (men vs women)	0.85	0.44 - 1.63	0.62
Fracture type (cervical vs subtrochanteric or pertrochanteric	0.92	0.52 - 1.63	0.77
Cardiovascular or cerebrovascular disease	2.78	1.32 - 5.88	0.01
Renal dysfunction*	1.04	0.54 - 2.00	0.91

*Defined as creatinine ≥105 umol/L in males and ≥95 umol/L in females

### Early postoperative complications

The total number of patients who suffered postoperative complications up to three months after the fracture did not differ between LdAA users and non-users (Table 4). But patients using LdAA preoperatively were significantly more likely to be afflicted with thromboembolic events, such as deep vein thrombosis and pulmonary embolism (5.7% vs. 0.7%, P = 0.03). Among LdAA users those who did not survive one year the number of patients with complications were 22 (63%) compared to the survivors 32 (39%).

**Table 4:** Early postoperative complications according to preoperative use of low-dose acetylsalicylic acid (LdAA)

Events	LdAA	No LdAA	P value
	(n = 118)	(n = 137)	
Cardiovascular	8 (6.8)	5 (3.6)	0.26
Cerebrovascular	2 (1.7)	0 (0)	0.13
Thromboembolic	6 (5.1)	1 (0.7)	0.03
Pulmonary infection	12 (10.2)	8 (5.8)	0.20
Lower urinary tract infection	23 (19.5)	25 (18.2)	0.80
Wound related	7 (5.9)	9 (6.6)	0.83
Reoperation	1 (0.8)	2 (1.5)	0.65
Other serious *	2 (1.7)	7 (5.1)	0.14
Any complication	54 (45.8)	48 (35.0)	0.08

* Kidney failure, gastrointestinal bleeding, septicemia (unknown origin), ileus, multiorgan failure, deep pressure ulcer.

## Discussion

The principal findings of our study were significantly higher need for blood transfusion postoperatively and higher first-year mortality in patients using LdAA before major hip surgery. The mortality risk in the preoperative LdAA users compared to non-users was increased nearly five times during the first month after hip fracture and was thereafter threefold higher up to one year after fracture.

Prophylactic treatment with LdAA for cardiovascular disease, both primary and secondary, is common in older people. An Irish study in 2006 showed that 42% of hip fracture patients were regular users of acetylsalicylic acid before the fracture [10]. Similarly, 46% of the patients in our study used LdAA, which can be compared to 17% of the whole population 80 years or older in Sweden during 2006 (according to the Swedish Prescription Drug Register).

At the time of our study national guidelines regarding prophylactic use of acetylsalicylic acid for cardiovascular disease recommended dosages between 75 mg to 320 mg but recent international recommendations have proposed a dose of 75 mg for most indications [11]. This can be of importance as most side effects of acetylsalicylic acid are dose-related and differences in effects on bleeding time and fibrinogenic activity have been shown with doses of 75 mg and 160 mg [12]. However, we did not find any significant difference in 1-year mortality between patients treated with ≤75 mg LdAA compared to those treated with ≥150 mg. Since acetylsalicylic acid in the blood is 50 to 80 percent protein-bound, metabolized for the most part in the liver and excreted mainly by the kidneys, the amount of active drug depends mainly on renal function and concomitant drug use [13]. Also, sex-related difference in excretion of acetylsalicylic acid has been shown, with women more likely to have higher drug concentrations in the blood [14]. Although the effect of preoperative use of LdAA on 1-year mortality in our study was higher in women than in men the difference was not statistically significant. This question may need to be investigated in a larger sample. Additionally, due to age-related decline in kidney function, higher concentrations of free salicylate can lead to increased risk of bleeding [15,16].

Preoperative discontinuation of LdAA in hip fracture patients is not an option as surgery should be done without unnecessary delay [17]. In doses of 75 to 160 mg irreversible blockade of cyclooxygenase lasts through the platelets' life span of 8 to 10 days. Because cardiac disease is common in hip fracture patients, withdrawal of LdAA for a few days may also increase the risk of cardiovascular complications[13]. Rebound phenomena connected to promotion of prothrombogenic factors is also a risk factor for thromboembolic complications which in itself makes withdrawal questionable [13].

In our original randomized trial we did not include patients with cervical fractures treated with internal fixation with hook pins, a less traumatic surgical procedure than hemiarthroplasty and usually not requiring blood transfusion. This group of patients usually includes both younger healthier patients and very old frail persons with limited walking ability or with an expected short life span. Because the three surgical procedures used in this study are major procedures that may differ in the amount of related blood loss we chose type of surgery rather than type of fracture in our analysis of bleeding and transfusion outcomes. However, using type of fracture in the analysis gave essentially similar results both in the original study and in this analysis (data not shown).

Both surgical and medical management of patients with hip fracture have improved during the last decade, but mortality has not decreased as might have been expected [3]. Despite that several studies have shown high mortality following hip fracture few studies have involved interventions to decrease it. The most frequently proposed solution has been to focus on fall prevention and not on interventions to diminish postoperative mortality. Doubts about the effectiveness of fall prevention on long-term mortality have been raised in a study comparing all-cause mortality in hip fracture patients with that of a general population [18]. Fields that need to be further studied include bleeding complications, blood transfusions, nutrition, congestive heart failure, hydration, chest infections, pharmaceutical treatment, cognitive function, and frailty.

Early mortality (within 120 days) following hip fracture has been shown to be related to patient age and sex, type of fracture, pre-fracture residence, mobility and ASA score [4,5,19]. A possible factor behind the higher mortality in the LdAA treated patients is the increased need for postoperative red blood cell transfusion. Patients treated with LdAA have increased bleeding diathesis and, in our study, they also had somewhat higher APTT and INR values, the reason for which is unclear because these values are not expected to be affected by LdAA. Although the differences are statistically significant, the numeric differences are small and their clinical relevance is uncertain. Other studies have shown up to 20 percent increase in blood loss in acetylsalicylic acid treated patients, but no significant increase in mortality [10,20]. Although some studies have shown doubled amount of bleeding complications in LdAA treated patients, the clinical significance of these complications has been disputed. Blood transfusions have been shown to both cause and to worsen congestive heart failure through circulatory overload and by causing fever, low blood pressure and pulmonary symptoms [10,21-23]. Studies showing significant increase in mortality in patients receiving transfusions have, however, mainly been carried out on patients in critical care units and not specifically on orthopaedic patients [23,24]. The 100 g/L hemoglobin level used as threshold for transfusion in our study may be considered to be relatively high. However, some previous studies have used a similar threshold although others have used a threshold as low as 85 g/L [20]. The indication for red cell transfusions should ideally be identified individually for each patient instead of a set pretransfusion threshold and be based on premorbidity and relevant clinical risk.

One reason for the higher mortality among the preoperative LdAA users may be the higher risk of early postoperative complications often leading to longer time before start of mobilization and rehabilitation [25]. Although postoperative complications were few there was a significantly higher incidence of thromboembolic events among LdAA users than among non-users. The number of patients afflicted with these complications was small but considering that LdAA treated patients had higher APTT and INR values and increased bleeding diathesis this seemingly paradoxical finding, merits further study. Even so it accentuates the need for a risk-benefit analysis when considering the use of antiplatelet therapy in frail elderly patients.

Previous studies of hip fracture patients have also shown an effect of preoperative LdAA on postoperative blood transfusion. Manning et al. studied 89 patients with femoral neck fractures treated with hemiarthroplasty or dynamic hip screw fixation with regard to the effect of preoperative aspirin on blood loss and transfusion requirements [26]. Although the authors showed no significant effect on perioperative blood loss, the 24 LdAA treated patients included in the analysis were significantly more likely to receive postoperative blood transfusion than the 52 patients without LdAA, a finding similar to our study, which also similarly did not show differences in intraoperative blood loss.

Some of the strengths of our study are that it included a representative sample of patients above 50 years with hip fracture, excluding only those with non-displaced cervical fracture treated with pin fixation (as those patients seldom require blood transfusion), that the majority of patients were operated within 24 hours after admission, and that we followed all participants prospectively for up to one year after surgery.

The main limitation of our study is that its primary objective was to evaluate the efficacy of a compression bandage in reducing need for blood transfusion and the mortality analysis is thus secondary. In the initial trial the proportion of LdAA treated patients in the two groups (compression vs non-compression) was similar and the compression bandage did not show any effects on postoperative blood transfusions or hemoglobin. We therefore assumed that the intervention with compression bandage did not influence the results of the present report. This is supported by the finding that adding postoperative use of compression bandage as a covariate to the analyses did not change the results. Another possible limitation is the large number of patients not assessed for eligibility for participation in the original trial by the orthopedic surgeon at the emergency room. Possible reasons for not assessing patients are that the surgeon did not know about the trial, had assessed the patients to be ineligible but failed to document this or was unwilling to recruit patients because of the extra work involved. However, these patients had similar characteristics to the patients included in the present report and since we have described the exact inclusion criteria for the trial we believe the patients in this study are representative of patients fulfilling these criteria.

Increased first-year mortality was seen both in patients using LdAA before fracture and in those with baseline cardiovascular and/or cerebrovascular comorbidity, with the latter having larger effect. The exact causes of the increased mortality among LdAA users are not known but this study does not exclude the possibility of side effects from acetylsalisylic acid as a contributing factor.

In a US study of 8930 patients with hip fracture it was shown that pulmonary complications were as common as cardiac complications in causing early mortality [27]. In our study only data on all-cause mortality were analyzed since we lacked information on cause of death. Finally, more detailed information on other medications used by the patients before surgery could have facilitated a more comprehensive assessment of the patients' risk of bleeding.

## Conclusions

In patients with hip fracture (cervical treated with hemiarthroplasty or trochanteric treated with internal fixation) preoperative use of LdAA was associated with significantly increased need for postoperative blood transfusions and significantly higher all-cause mortality during one year after surgery.

## Competing interests and funding

The authors have no competing interests to declare. The project was supported by independent research grants from Skåne County Council's Research and Development Foundation, the Swedish Society of Medicine and Hässleholm Hospital. The grant providers had no role in study design, data collection, statistical analysis, or manuscript preparation and submission. No financial or other support from other sources was received.

## Authors' contributions

Conception and design of the study: AK, MW, AA, IA. Data collection: AA, AK, MW. Statistical analysis and interpretation: IA, PW, AK. Drafting of the manuscript: AK, IA, MW. Critical revision of the manuscript: MW, AA, PW. Approval of the final version of the manuscript: AK, MW, AA, PW, IA

## Pre-publication history

The pre-publication history for this paper can be accessed here:

http://www.biomedcentral.com/1471-2474/12/254/prepub
